# Associations among positive childhood experiences, resilience, and psychological distress in health professions students

**DOI:** 10.1080/10872981.2026.2694936

**Published:** 2026-06-26

**Authors:** ThanhThanh Vo, Jeffrey Duong, Cara Sandholdt, Elizabeth Rice, Karl Jandrey, Margaret Rea, Michael Wilkes, Andrés F. Sciolla

**Affiliations:** a University of California, San Francisco – Fresno, Department of Psychiatry, Fresno, CA, USA; b Healthy Rural California, Chico, CA, USA; c University of California, Davis, Betty Irene Moore School of Nursing, Sacramento, CA, USA; d University of California, Davis, School of Veterinary Medicine, Davis, CA, USA; e University of California, Davis, School of Medicine, Sacramento, CA, USA

**Keywords:** Positive childhood experiences, health professions students, resilience, psychological distress, mediation analysis

## Abstract

**Objective:**

To assess the mediating role of psychological resilience in the negative association between positive childhood experiences (PCEs) and psychological distress in health professions students (HPS).

**Methods:**

The authors obtained cross-sectional data, including demographic information, the Brief Resilience Scale, and Medical Student Well-Being Index, from a longitudinal survey with circannual waves of data collection in medical, advanced practice provider, (i.e., nursing and physician assistant), and veterinary students at a single institution. The confidential, web-based survey included seven questions about PCEs. The authors conducted a mediation analysis of wave 3 of the data (*N* = 181) using structural equation modeling, which adjusted for age, gender, underrepresented in medicine (URM) status, and first-generation status. Bootstrap confidence intervals modeled and tested the total and indirect pathways.

**Results:**

The path model demonstrated an excellent fit with the data (CFI/TLI = 1.00; RMSEA < .001; SRMR < .001). As hypothesized, PCEs were positively associated with resilience (b = 0.020; *p* = .049), and resilience was negatively associated with psychological distress (b = −0.815; *p* = .003). Although PCEs were negatively associated with psychological distress (b = −0.061; *p* = .003), the indirect path between PCEs and psychological distress through resilience was not significant (b = −0.016; *p* = .114).

**Conclusion:**

To our knowledge, this is the first study to examine PCES and resilience in HPS. PCEs and resilience may represent independent predictors of mental well-being in HPS. Our findings underscore the significance of identifying students with fewer PCEs and adopting interventions that support the psychological well-being of HPS, especially those who might be at a higher risk of distress.

As essential contributors to the future of healthcare, health professions students (HPS) are integral to shaping and sustaining a robust healthcare system. However, studies suggest that these students often experience high rates of psychological distress and burnout [1]. Other research has shown that psychological distress may be associated with negative sequelae among medical students, such as poor academic performance and unprofessional behaviours [2,3]. Arguably, the effectiveness of interventions that target psychological distress depends on a comprehensive identification of its most significant drivers, both direct (e.g., academic burden) and indirect (e.g., those conferring risk by interacting with direct drivers). Among the latter, adverse childhood experiences (ACEs) have been identified in multiple groups, including medical students, as robust drivers of distress [4]. Comparatively little is known about the potential influence of positive childhood experiences (PCEs), a reciprocal measure of ACEs including supportive relationships and environment, on HPS health and well-being [5,6]. This is critical gap, considering evidence in other populations suggesting a dose-response relationship between PCEs and better mental health, independent of ACEs exposure [5,7]. Furthermore, PCEs may be associated with positive outcomes *directly*, in addition to potentially moderating the effects of adversity on outcomes [7]. The mechanism of the PCEs-mental health association is incompletely understood, but there is growing evidence suggesting that psychological resilience has a mediating role in mental well-being [8,9], sleep disturbance [10], life satisfaction [11], and flourishing [12]. Resilience, the ability to ‘bounce back’ in the face of adversity, has increasingly been recognised as an essential skill for HPS and practitioners to survive and thrive in their stressful endeavours [13]. In contrast to unchangeable ACEs and PCEs, resilience can be enhanced in these groups, which result in lower levels of anxiety and perceived stress [14].

To our knowledge, this study is the first to address the paucity of data on the relationship between PCEs, resilience, and psychological distress in HPS. Specifically, this study aims to assess resilience as a mediator in the association between PCEs and psychological distress among HPS. We hypothesised that PCEs would be associated with greater levels of resilience among HPS, which in turn would be associated with a lower likelihood of psychological distress. Second, we hypothesised that resilience would mediate the relationship between PCEs and psychological distress among HPS. HPS face a demanding, fast-paced curriculum, under high pressure [15]. Thus, the findings from this research may offer insight into potential intervention strategies to enhance the mental well-being of these students.

## Method

The data were obtained from a longitudinal survey of HPS enroled in 2019 at a single institution, including medical, advanced practice provider (APP), nurse practitioner and physician assistant, and veterinary students. At matriculation (wave 1), all newly 357 matriculated students were invited to participate by email by each school’s Dean of Students and a designated data manager. A total of 261 eligible students consented and completed the baseline study. The students comprised those enroled in 2-year master-level NP and PA programs, while medical and veterinary students enroled in 4-year doctoral-level programs. Participants completed confidential, web-based questionnaires through Qualtrics survey software (Qualtrics, Provo, UT) and were incentivized with a small monetary gift. The baseline survey was open for a month in 2019, with up to three weekly email reminders sent to non-responders. Only students who completed the baseline survey were invited to complete successive follow-up yearly surveys. With participant consent, survey responses were linked to demographic and academic records. In total, the sample at wave 1 comprised 240 HPS, including 104 medical and 89 veterinary students, who completed five survey waves over four years, and 47 APP students, who completed three successive surveys over two years. The study was approved by the Institutional Review Board.

The participants' sociodemographic characteristics, including gender, first-generation status, and race/ethnicity, were provided by the registrar's office. Students were categorised as ‘underrepresented in medicine (URM)’ if they identified their race/ethnicity as belonging to any group except white or Asian.

The survey included 12 brief, validated self-report measures assessing individual, group, and institutional variables associated with resilience and psychological distress. Details of the study’s theoretical framework have been published elsewhere [16]. In the present study, we focus on data collected at wave 3 (*N* = 131), which included a PCEs questionnaire in light of the growing evidence of the associated between PCEs and multiple health outcomes, including resilience [5]. Since the questionnaire refers to (unchangeable) experiences before age 18, it was not necessary to include it in other data collection periods.

The 6-item Brief Resilience Scale (BRS) was used to measure resilience (Cronbach's alpha = 0.80 to 0.90) [17]. The measure employs a 5-point Likert scale for statements such as ‘I tend to bounce back quickly after hard times.’ Participants' responses were summed for the six items and then divided by the total number of questions answered, yielding a score range from 1 to 5. Higher average scores were indicative of greater resilience. Scores 3.00–4.30 indicate normal resilience, while 1.00–2.99 and 4.31–5.00 scores indicate low and high resilience, respectively [17].

The Medical Student Well-Being Index (MSWBI) is a 7-item tool that identifies students at risk for psychological distress (Cronbach's alpha = 0.68) [18]. An index score of four or greater has demonstrated a sensitivity and specificity of greater than 90 percent for identifying students with low mental quality of life or recent suicidal thoughts or thoughts of dropping out of school [18]. The measure has been used in previous studies to study the well-being of non-medical HPS, such as pharmacy students, as well as non-HPS populations, such as nurses [19,20]. A 2019 research study also showed the efficacy of this index in nurse practitioners and physician assistants [21].

The positive childhood experiences (PCE) measure comprises 7 items that enquire about the frequency of respondents' childhood experiences. Questions query about the extent respondents felt while growing up [1] able to talk to their family about feelings; [2] their family stood by them during difficult times; [3] enjoyed participating in community traditions; [4] a sense of belonging in high school (not including those who did not attend schools or were homeschooled); [5] supported by friends; [6] had at least 2 nonparent adults who took genuine interest in them; and [7] safe and protected by an adult in their home. These items were adapted from the Child and Youth Resilience Measure–28 and were designed to ensure cultural sensitivity [5]. Psychometric analyses affirmed the use of a cumulative PCEs score (Cronbach's alpha = 0.77).

We conducted a simple mediation analysis with cross-sectional data in R software (v 4.3.2; R Foundation for Statistical Computing) using structural equation modelling (SEM) with the Lavaan package 0/0/00 0:00:00 AM [22]. SEM allows multiple path coefficients in a hypothesised model to be estimated simultaneously. Model fit was assessed using the comparative fit index (good fit > 0.95), Tucker-Lewis Index (good fit > 0.95), root-mean-square error of approximation (RMSEA; good fit < 0.05), and standardised root mean squared residual (SRMR; good fit < 0.09) [23]. Analyses were adjusted for age, gender, URM status, and first-generation status. Total and indirect pathways were tested with bootstrap confidence intervals. Regarding missing data, the quality of the dataset is such that missing responses were infrequent for each of the individual survey items (less than 5%) [24]. Moreover, values were determined to be missing at random. In our analysis, we used full information maximum likelihood to compute estimates using all available data [25,26].

## Results

The characteristics of the sample can be found in Table 1. Out of 240 participants, 80.4 percent of our participants were female, 21.9 percent were URM students, and 22.4 percent were first-generation HPS. The mean age for the participants in this study is 24.8 years (SD = 4.8).

**Table 1.** Sample Characteristics (*N* = 240).

	Mean (SD*) or *n* (%)
Mean Positive Childhood Experiences Score (out of 35)	26.5 (5.2)
Mean Resilience Score (out of 5)	3.5 (0.6)
Mean Psychological Distress Score (out of 7)	3.7 (2.0)
Female	115 (80.4)
Underrepresented in Medicine (Not White/Asian)	30 (21.9)
Mean Age (Years)	24.8 (4.8)
First Generation Student	32 (22.4)

Note: SD = Standard Deviation

**Figure 1.**
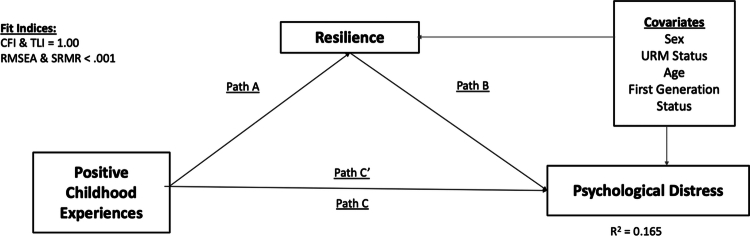
The model results of PCEs, Resilience, and Psychological Distress. Path A, B, and C’ represent the association between the two variables; total and indirect pathways were tested to analyse the mediating role of resilience in the relationship between PCEs and psychological distress. Path A: b = 0.020 (*p* = 0.049), 95% CI = 0.002, 0.043. Path B: b = −0.815 (*p* = 0.003), 95% CI = −1.363, −0.276. Path C’: b = −0.061 (*p* = 0.003), 95% CI = −0.118, −0.009. Path C: Total Effect = −0.077 (*p* = 0.004), 95% CI = −0.131, −0.025. Indirect Effect = −0.016 (*p* = 0.112), 95% CI = −0.041, −0.001.

The model results are shown in Figure 1. The path model demonstrated an excellent fit with the data (CFI/TLI = 1.00; RMSEA < .001; SRMR < .001). The reported coefficients are unstandardised and therefore reflect changes in the outcome variable per one-unit change in the predictor according to its original scale. As hypothesised, PCEs were positively associated with resilience (path A) (b = 0.020; *p* = .049; 95% Confidence Interval [CI] = 0.002, 0.043). Although significant, this effect was small in magnitude. Resilience was negatively associated with psychological distress (path B) (b = −0.815; *p* = .003; 95% CI = −1.363, −0.276), indicating a moderate and meaningful protective effect of resilience in relation to psychological distress. PCEs were also negatively associated with psychological distress (path C’) (b = −0.061; *p* = .003; 95% CI = −0.118, −0.009), representing a small but practical effect.

Based on the results, the total effect (path C = a*b + c’) shows that overall, students with higher PCEs reported lower psychological distress (b = −0.077; *p* = .004). However, the indirect path (A*B) between PCEs and psychological distress through resilience was not significant (b = −0.016; *p* = .114), indicating that resilience did not mediate the relationship between PCEs and distress.

## Discussion

While there is a plethora of research on the detrimental effects of ACEs on mental well-being, little is known about the role of PCEs in alleviating psychological distress among HPS and the potential role of resilience as an individual’s psychological strength in mediating this relationship [27]. Our study conceptualises distress and resilience as shaped by the interaction of variables across multiple levels, including institutional (e.g., the learning environment) and structural (e.g., daily discrimination), in addition to intrapersonal influences [16]. Measures of learning environment and discrimination were included in the longitudinal study and will be the focus of future manuscripts. Our conceptualisation of resilience as a multilevel construct also aligns with critiques of interventions that focus solely on strengthening individual resilience among HPS [28,29]. In this cross-sectional analysis, we found a positive association between PCEs and resilience and a negative association with psychological distress among HPS. Contrary to our hypothesis, resilience did not mediate these relationships. Nevertheless, we suggest that our findings can inform the development of interventions for students enroled in health professions pathway programs, as well as for interventions seeking to enable current trainees to cope more effectively with stressors and minimise psychological distress.

The positive association between PCEs and resilience we found is consistent with prior research [5,27,30]. Importantly, we found that students with higher PCEs reported lower levels of psychological distress as adults and under notoriously stressful academic pursuits [31,32]. Although this association between PCEs and lower psychological distress has been observed across the lifespan of adults in the general population [5], we observed this effect in a sample of HPS who are at heightened risk for mental health problems with serious academic and personal consequences [1,2]. This study extends the scientific literature by demonstrating the potential protective contributions of PCEs to HPS’ psychological well-being a decade or more later and suggests interventions that leverage students’ strengths instead of narrowly focusing on ACEs and deficits [33,34].

Given the potential associations of PCEs with lower adverse outcomes related to ACEs as well as better health outcomes, clinicians and policymakers have been encouraged to identify and promote PCEs in children, adolescents, and adults [6,35,36]. Empirical evidence of effectiveness, however, is only beginning to emerge in children [37]. We are not aware of published interventions in college students or HPS leveraging PCEs to enhance well-being and reduce psychological distress. Future research in this area may offer valuable insights and be congruent with strengths-based interventions that are supported by experimental [38,39] and qualitative studies [40].

Taken together, our findings and others highlight the potential relevance of PCEs in the mental health of HPS. High PCE scores highlight the presence of positive childhood resources that can nurture resilience, as demonstrated by a study of Chinese nursing students that found a dose-response association of PCEs with meaning in life and flourishing independent of perceived stress [12]. The authors conclude with recommendations for institution-based screening of PCEs and targeted interventions for high- and low-scoring students [12]. Because low PCE scores point out resources that were absent but needed during students’ formative years and the likelihood of ACEs exposure, universally applied trauma-informed pedagogy may benefit students reporting low PCEs [41,42]. At the same time, this approach may avoid the potential stigma associated with screening for ACEs outside a confidential setting (e.g., counselling session, primary care provider visit) [43,44].

An additional implication pertains to outreach and pathways programs. Assessing PCEs can identify students who may benefit from coping skills interventions [45] or require more additional support [46]. Institutions can further promote resilience through extracurricular engagement and mentoring—approaches that mirror the PCEs that build resilience in childhood [47,48].

Our findings showed that HPS with higher resilience scores were less likely to report distress. While this study did not assess how resilience can be enhanced by specific interventions, the findings suggest that resilience could serve as a target for schools’ wellness programs to identify students who might be at a higher risk for distress [49]. They can consider prioritising students’ well-being by providing and expanding therapy sessions [50] and tailor assistance and academic support services based on students’ needs and strengths [51]. Current literature underscores the critical roles of institutional support and academic climate in student wellness, demonstrating that low faculty support is independently associated with severe distress, and the absence of mentorship increases its odds by 63% [52]. The learning environment shapes resilience through multiple ways, including but not limited to academic workload and access to support systems [52].

We acknowledge that resilience-based interventions have been critiqued for placing the burden of strengthening resilience on individuals rather than addressing the systemic and structural conditions that might cause distress in the first place [28,49,50]. Others have pointed out that health professions schools should be responsible for creating equitable and supportive learning environments and addressing how students experience school rather than encouraging healthy practices outside of it [28,53]. When identifying students with disadvantaged backgrounds for targeted support, institutions must do so through ethically grounded frameworks that prioritise student autonomy and avoid stigmatisation and implicit bias, which can discourage students from utilising resources that they need [54]. Screening and outreach efforts should be voluntary, culturally responsive, and transparent about how information will be used when connecting students to resources and support networks. Since psychological distress is not confined to students with low PCEs, we recommend that school administrators engage in strengths-based and trauma-informed approaches through the expansion of psychological services and academic support tailored to students’ specific needs and learning styles regardless of their backgrounds.

We found that resilience did not mediate the relationship between PCEs and the degree of distress experienced by HPS. One possibility for the non-significant finding could be the minimal variability in resilience scores within our sample, which consisted of relatively highly resilient individuals. Low variability might mitigate the ability to detect the mediating role of resilience in mediation models. Additionally, resilience in this population might act more as a stable trait rather than a dynamic mechanism through which early positive childhood experiences influence psychological distress. However, PCEs and resilience remained as significant predictors of psychological distress in our path model, highlighting that they function as independent, rather than sequential, predictors of psychological distress among HPS.

Notably, our study began in 2019 and continued through the COVID pandemic

However, longitudinal studies and meta-analyses reaffirm that PCEs and resilience maintain the predictive validity of both pre- and post-COVID [7,55,56]. While the overall psychological stress might have been elevated during the pandemic, the associations between PCE, resilience, and distress persist over time and are independent of external stressors [57].

The cross-sectional analysis limits our ability to examine whether there is a temporal relationship between PCEs, resilience, and psychological distress among students. All variables were assessed at a single time point, so we cannot establish the directionality of these associations or confirm temporal pathways. Furthermore, the retrospective self-reporting of PCEs introduces recall bias, as participants’ current psychological states might influence how they remember and report their childhood experiences. Therefore, there is a need for a longitudinal analysis across multiple institutions to fully understand the mechanisms at play in this specific population. Selection bias might also be present because our study might have attracted students with particular characteristics or levels of psychological distress. The questionnaires may be subject to ascertainment bias, as some participants who are more conscious of mental health may have been more likely to complete the surveys. Attrition may bias findings as well, since participants who drop out over time may have higher psychological distress and lower resilience, which could result in a remaining sample that appears healthier than the original cohort. Our findings were collected from a single institution and the majority of our participants identified as female, which might limit the generalisability of our findings and not represent all HPS in the United States. Conversely, our findings might be influenced by the diverse personality traits of our participants, which includes nurse practitioner, physician assistant, veterinary medicine, and medical students [58]. For example, higher empathy in nursing compared to medical students [59] may obscure associations among the study variables, such as PCEs and empathy [60]. Veterinary medicine students might have different motivational profiles compared to human medical students [60]. In our study, we did not examine the differences across trainee groups. As such, it is possible that the relationships observed in this study may differ across HPS. Future research should assess whether resilience functions as a protective factor across distinct HPS populations. Qualitative research methods (e.g., interviews and focus groups) can afford an in-depth understanding of mental health and school experiences of HPS. Finally, future investigations should target students in other health professions schools (e.g., dentistry, pharmacy) to ascertain whether these variables might yield similar results or our findings.

In conclusion, our study contributes to current research studies by demonstrating a positive association between PCEs and resilience, and a negative association between PCEs and psychological distress. Notably, in our sample of highly resilient HPS, PCEs and resilience functioned as independent predictors of psychological distress. Second, HPS with higher resilience levels reported less psychological distress, which suggests that resilience might play a protective role as HPS navigate the demands of rigorous training. These findings underline the importance of fostering resilience in HPS to enhance their ability to adapt to academic and clinical challenges during training. Third, our result highlights that HPS growing up in environments with more PCEs were associated with less psychological distress during their training. Finally, our findings suggest that resilience might not significantly mediate the association between PCEs and psychological distress, which serves as a valuable reminder that it might not be enough for students to cultivate resilience on their own. Institutions bear equal responsibility for cultivating equitable, trauma-informed learning environments that support all students. We urge schools to move beyond self-care education toward ethically grounded, strengths-based approaches that prioritise universal access to mental health and academic support. The approaches should also prioritise early pathway programming for disadvantaged adolescents interested in pursuing health professions. This will help cultivate a new generation of healthcare professionals grounded in kindness and compassion, while equipping them to meet the psychological demands of intensive health professions education programs.

## Data Availability

The datasets generated and/or analysed during the current study are not publicly available due to participant confidentiality but may be available from the corresponding author on reasonable request.
